# The Investigation of Cognitive Functions and Clinical High Risk Status for Psychosis in First-Degree Relatives of Patients with Substance Induced Psychotic Disorder

**DOI:** 10.1192/j.eurpsy.2022.533

**Published:** 2022-09-01

**Authors:** M. Çukurova, A. Özdemir

**Affiliations:** 1 Erzurum Hınıs Şehit Yavuz Yürekseven Devlet Hastanesi, Psychiatry, Erzurum, Turkey; 2 Privat Office, Psychiatry, Istanbul, Turkey

**Keywords:** Clinical high risk groups, Familial predisposition, Substance induced psychotic disorder, Neurocognitive function

## Abstract

**Introduction:**

The etiology of substance-induced psychotic disorder (SIPD) is an important research area to study.

**Objectives:**

It is aimed to investigate clinical risk status for psychosis, schizotypal features and neurocognitive functions in siblings of the patients who have been diagnosed as SIPD and who have no family history of psychotic spectrum disorder.

**Methods:**

This study included 41 healthy siblings of patients who have been diagnosed as SIPD according to DSM-V and 41 healthy controls without family history of psychiatric disorders (matched on age, gender, and years of education). The data collected with sociodemographic and clinical data form, Digid Span Test, Trail Making Test A, Trail Making Test B, Verbal Fluency Test and Stroop Test, Comprehensive Assesment of At-Risk Mental States (CAARMS) and Structured Interview for Schizotypy-Revised.

**Results:**

It is determined that %41.5 of siblings and %7.3 of healthy controls are in one of the clinical high risk groups for psychosis according to CAARMS. There is significant difference in Trail Making Test A error and Trail Making Test B error and correction, verbal fluency test- lexical fluency-perseveration mean scores between siblings of patients and healthy controls.

**Figure:**
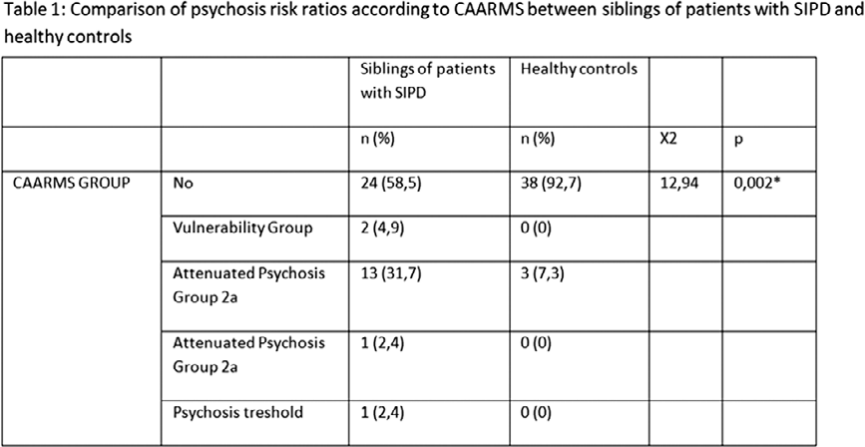

**Conclusions:**

Siblings of patients with SIPD have more schizotypal features than healthy control group and they take part more frequent in one of high risk group for psychosis. Schizotypal features are known as trait factor and show genetic predisposition. Siblings who are in high risk groups have more schizotypal features and it may point that predisposition to psychosis is more related to underlying genetic predisposition than environmental factors and social stressors.

**Disclosure:**

No significant relationships.

